# A Few Words about Homœopathy

**Published:** 1872-02

**Authors:** 


A few Words about Homœopathy.—Among the laity, an impression very generally prevails that the objection of the "old school" to homœopathy is mere prejudice, and that we are only prevented by an absurd punctilio from consulting with those who practice it; and such is really the case, unless we know what the doctrines are which we decline to recognize. We do not hesitate to say that the profession should, one and all, be well acquainted with this system of quackery—its apparently strong points, and its really weak ones.

We have no fear that familiarity with the ground-work and the current literature of homœpathy will be a source of danger to any well-educated or honest practitioner of regular medicine. At the present day, after more than thirty years, a profession composed of a large body of men of more than average education, ever in eager search for new light upon the causes of human suffering, and the remedies therefor, have utterly failed to find in homœpathy anything to aid them in their battle with disease. Not one man of note in our ranks has come out and announced himself as either in whole or in part a convert to that doctrine. Not one physicist of eminence has ever given in his adhesion to its principles, or recognized it as the offspring of true science. Not one theory or fact, directly or indirectly developed by homœpathy, has worked its way into acceptation either by men of general science or by those devoted to medicine especially.

We cannot consult with homœpaths, because we have no common ground upon which to meet them in the discussion of medical matters. Our code of ethics most justly prohibits us from consultation with all those "whose practice is based upon an exclusive dogma, to the rejection of the accumulated experience of the profession, and of the aids actually furnished by anatomy, physiology, pathology, and organic chemistry." No medical society can be compelled to admit them as members, nor can any legal penalty be enforced against those who decline to recognize and consult with them (as has recently been attempted in Massachusetts), except under laws so absurd as to work their own abrogation.

Homœpaths themselves—unless they are grievously slandered by report—in desperate cases, turn their backs upon their dogma, abandon their infinitesimal doses, their triturations and potencies, and resort to practice which would be "heroic" if it were not too often reckless and hurtful.

We say, again, that not only honesty, but interest, would induce us—would compel us—to adopt whatever of truth there was in homœpathy, if it contained such an element. We assert that it cannot stand the test of impartial scientific scrutiny, and that its existence is owing to the fact that before its patrons such test can never be applied. It can, however, be done by every member of the profession for himself; so that he may be, instead of an ignorant and bigoted opponent, an intelligent and candid judge of a system of quackery whose semblance of science alone raises it above other forms of charlatanism.—*Philadelphia Medical Times.*

